# One More Unfortunate

**Published:** 1867-08

**Authors:** 


### "ONE MORE UNFORTUNATE."

An indiscreet friend is worse than a foe—worse than *two* foes. When the man that stood god-father for Lamm's gold at the Boston meeting of the American Dental Association urged, as one of its chief claims to favor, that it could be used *successfully* under the saliva, we felt sorry. The gold is useful; but no man could have done so much against it by the bitterest denunciation as this *friend* did by this preposterous claim. We know it can be pressed into a cavity, so as to look like a fair filling, in spite of moisture. We prepared that kind of gold as early as 1853. But it is unfortunate when operators are thus coaxed into negligence in regard to an essential element of good operating—essential, because *gold is gold.* Again, allow us to say we were sorry to see this preparation thus wounded in the house of its friends.

Somebody's "plastic gold" was alike unfortunate at the recent meeting of the Association in Cincinnati. An ordinary cavity could be filled with it in less than two minutes—"in a whisper." When this gold is pressed, by an obtuse instrument, into retaining grooves or pits, the projecting portions cannot be pressed down with a burnisher; but when the external surface of the filling is thoroughly condensed, inequalities can be burnished down with ease. In camp-life we sometimes wondered how it came that the boards always lay with the *hard side* uppermost. This gold is more accommodating. If you wish to file or burnish it, it turns its soft side to the instrument. If you desire to fasten it in a badly shaped cavity, it "sticks in its holders," and hardens its heart, like Pharaoh of old. But we are not able to give expression to even an average specimen of the peddlers' twaddle with which it was beslimed by the half hour. We write this to tell our readers that the "plastic gold" is a pretty good thing, in spite of that speech, and to advise them to give it a fair trial.

When Sut Lovengood's daddy was "actin' hoss," he got into a nest of hornets, and jumped over a precipice into a mill-pond. Sut thought "if he meant that for actin' he rather overdid it; a mule might have done that; but dad wa'n't actin' mule." It is unfortunate that some still fall into the mistake of "Lovengood" senior.

